# Machine Learning Algorithms for Automatic Classification of Marmoset Vocalizations

**DOI:** 10.1371/journal.pone.0163041

**Published:** 2016-09-21

**Authors:** Hjalmar K. Turesson, Sidarta Ribeiro, Danillo R. Pereira, João P. Papa, Victor Hugo C. de Albuquerque

**Affiliations:** 1 Instituto do Cérebro, Universidade Federal do Rio Grande do Norte, Natal, Brazil; 2 Departamento de Computação, Universidade Estadual Paulista “Júlio de Mesquita Filho”, Bauru, São Paulo, Brazil; 3 Programa de Pós-Graduação em Informática Aplicada, Laboratório de Bioinformática, Universidade de Fortaleza, Fortaleza, CE, Brazil; Texas A&M University College Station, UNITED STATES

## Abstract

Automatic classification of vocalization type could potentially become a useful tool for acoustic the monitoring of captive colonies of highly vocal primates. However, for classification to be useful in practice, a reliable algorithm that can be successfully trained on small datasets is necessary. In this work, we consider seven different classification algorithms with the goal of finding a robust classifier that can be successfully trained on small datasets. We found good classification performance (accuracy > 0.83 and F_1_-score > 0.84) using the Optimum Path Forest classifier. Dataset and algorithms are made publicly available.

## Introduction

The common marmoset (*Callithrix jacchus*) is a species of arboreal New World monkeys native to the northeast region of Brazil. This species is becoming an increasingly important primate model of a number of human diseases, as well as for basic research in neuroscience [1, 2] and genetics [3]. Examples of the species’ use as a disease model come from multiple sclerosis [4], herpes virus [5] and tuberculosis [6] research. Some of the reasons for this popularity are that marmosets have a similar disease susceptibility profile to humans, are relatively easy to handle, have a high reproductive rate, and that important genetic and neuroscience research tools already exist [7]. In particular, marmosets are an excellent model for the neurophysiological study of vocal communication [8]. A pubmed search on “Callithrix jacchus” shows between 113 and 164 publications per year during the last 10 years, with most of the publications in biomedicine. Given the widespread use of marmosets in laboratories, methods to reliably monitor health and behavior in captive colonies are of a high priority.

As typical for Neotropical arboreal primates, marmosets rely heavily on acoustic communication. This together with cooperative breeding and the complex social organization that follows, means that marmosets have a large vocal repertoire, with 9–13 different types of vocalizations reported [9–13]. The vocalizations produced by a group of marmosets provide a rich source of information about their activities and well-being [14]. However, although informative, it is practically untenable to manually track the vocalizations of a colony over any longer period of time. Manually classifying calls require expert knowledge and daily hours of work, making it too time-consuming and prone to inconsistencies among researchers. Thus, a reliable automatic method for call identification is necessary.

Much work has been done on the automatic classification of animal vocalizations, especially bird song [15–19], but also mammalian [20–22] and amphibian calls [23–25]. However, only a few studies have addressed non-human primates (hereafter primates). Pertinent to the current study, there are only three other studies in which different primate vocalizations were analyzed. Mielke and Zuberbühler (2013) used artificial neural networks (ANNs) with Mel-Frequency Cepstral Coefficients features to classify blue monkey call types (*Cercopithecus mitis stuhlmanni*). Furthermore, they predicted caller identity from the alarm call, and identified the blue monkey alarm call among the alarm calls of other sympatric species [26].

Pozzi, Gamba and Giacoma (2010) also used ANNs, but with hand-designed features derived from fundamental frequency and formants to classify call type among seven distinct types made by the black lemur (*Eulemur macaco*) [27]. In a later study, the same group used the long grunt (a vocalization included in the repertoire of all lemurs) and similar analytical methods to classify species among the five species in the *Eulemur* genus [28]. Therefore, to our knowledge, there is no previous work on classifying vocalization types from Neotropical primates.

In contrast, several studies have explored the related question of how specific to the caller are the acoustic properties of individual vocalizations, that is, how well caller identity can be predicted from a given call. These studies have all relied on manually designed features and linear discriminant analysis (LDA). Particularly relevant are Jones et al. (1993) and Miller et al. (2010), who both studied the common marmoset’s Phee call to classify caller individual [29, 30]. Both studies found that the call is highly caller-specific. Similar studies have been done on Japanese macaque (*Macaca fuscata*) [31], blue monkey [32], ring-tailed lemur (*Lemur catta*) [33], and cotton-top tamarin (*Saguinus oedipus*) [34].

Algorithms for the automatic classification of vocalizations learn the mapping from input (call features) to label (call type). Therefore, a dataset with labeled calls is necessary to train the algorithms. In general, classification performance increases with the amount of training data. However, in ethology, large sets of labeled data are often hard to obtain. The amount of data may be limited because data collection is labor-intensive, or the data of interest are inherently scarce because they are produced by animals passing through transitory learning or developmental stages. In the latter case, the amount of possible data is strictly limited. Thus, a method that achieves high accuracy with a relatively small number of labeled call exemplars is highly desirable.

To address the need for automatic call classification given the aforementioned constraints, we compared seven different types of classification algorithms with the goal of finding a reliable method.

To extract acoustic features, we used Linear Predictive Coding (LPC), a method commonly used for speech processing [35]. For classification purposes, we used seven different algorithms: (i) Optimum Path Forest (OPF), (ii) Bayesian Classifier, (ii) Multilayer Artificial Neural Network (MLP), (iv) Support Vector Machines (SVM), (v) k-Nearest Neighbors (k-NN), (vi) Logistic regression, and (vii) AdaBoost.

## Materials and Methods

### Dataset description

The subjects were five captive-born adult common marmosets (two females and three males), housed at the Instituto do Cérebro, Universidade Federal do Rio Grande do Norte. The marmosets where housed socially in two wire mesh enclosures (1.20 x 1.50 x 2.45 m), enriched with tree branches, ropes, plants, hammocks and nesting tubes. The animals were fed twice daily with fresh and dried fruit, nuts, egg and chicken, and had *ad libitum* access to water. The colony was maintained outdoors protected by a roof allowing daily sunbaths in natural light. The animals were housed in compliance with SISBIO permit 18394, and the experiment was approved by the ethics committee of Universidade Federal do Rio Grande do Norte with CEUA permit 11/2016. No animal was sacrificed at the end of the experiment.

A directional microphone (ECM-CG50 Pro Shotgun Microphone, Sony, Tokyo, Japan) was placed at a distance of 10 cm above the home cage and connected to a computer. The microphone signal was streamed to a computer where a custom-written Python script segmented the incoming signal. The signal was bandpass filtered between 4 and 10 kHz, and when the amplitude exceeded a threshold a segment beginning 0.5 s before first threshold crossing and ending 0.5 s after the last threshold crossing was saved. Sound was sampled at 44.1 kHz.

**Fig 1:**
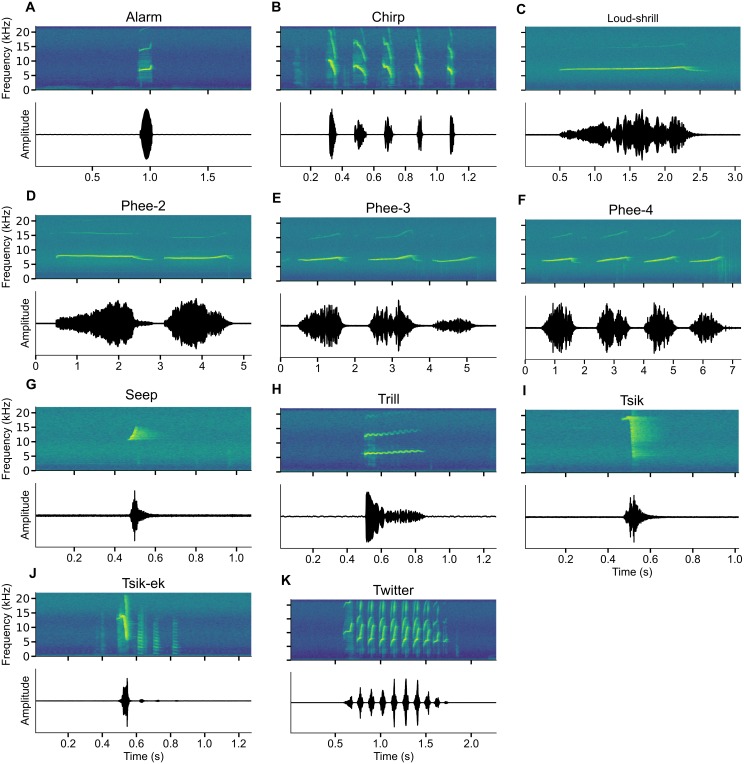
Vocalization exemplars. Amplitude and time-frequency spectrograms are shown for representative exemplars of the marmoset call types considered in this study. A: Alarm, B: Chirp, C: Loud shrill, D: Phee-2, E: Phee-3, F: Phee-4, G: Seep, H: Trill, I: Tsik, J: Tsik-Ek, K: Twitter.

From the raw recordings, we selected and manually labeled 27–30 exemplars per class of marmoset vocalization. We attempted to cover the marmoset’s vocal repertoire of approximately nine [36] to 13 [37] distinct vocalizations. The number of exemplars of each of the 11 types investigated in this work is listed in Table 1, and spectrograms of representative exemplars of each type are shown in Fig 1. All vocalizations were produced spontaneously, that is, without any intervention from the experimenter. The recordings were done over a period of two months. The dataset is available at https://osf.io/yqpvk/ and http://neuro.ufrn.br/data/marmosetvocalizations.

**Table 1:** The number of calls considering each class.

Vocalization type	Exemplars per class
Alarm	30
Chirp	30
Loud-shrill	27
Phee-2	30
Phee-3	30
Phee-4	30
Seep	30
Trill	27
Tsik	27
Tsik-ek	30
Twitter	30

### Feature extraction

The success of a signal classification system depends on the choice of features used to characterize the raw signals. In this work, we used LPC, a method commonly used for analysis and compression of speech and animal vocalizations [35, 38]. The linear prediction filter coefficients were used as input features to the classification algorithms. Those are the coefficients of an n^th^-order linear finite impulse response filter that predicts the current value of the vocalization from past samples [39]. The number of features extracted from each call was set to 20 after experimenting with filter orders from 10 to 25, in steps of 5.

### Classification algorithms

#### Optimum-Path Forest

The OPF classifier models the problem of pattern recognition as a graph partition task, in which a predefined set of samples from each class (i.e. *prototypes*) compete for minimal path cost to the rest of the samples. This results in a collection of optimum-path trees rooted at the prototype nodes, building an optimum-path forest considering from all training samples. Test samples are classified through incrementally evaluating the optimum paths from the prototypes, as though they were part of the forest, and assigning the labels of the most strongly connected roots. The notion of optimum-path connectivity comes from the minimization of a path-cost function [40]. An OPF classifier can be designed as long as we use a smooth path-cost function. Although there are two different versions of the supervised OPF classifier [41–43], in this paper we make use of the former and most widely used approach, as described below.

The OPF with complete graph was first proposed by Papa et al. [41]. Later on, Papa et al. [42] presented an improved version with a more efficient classification step. In this section, we present the OPF algorithm as described by those authors, using the same formalism.

Let $D=D_{1}\cup D_{2}$ be a dataset partitioned into a training ($D_{1}$) and a test ($D_{2}$) set. In addition, let G=(V,E,w) be the graph originated from $D_{1}$, such that $V=D_{1}$ and E stands for an adjacency relation that defines a full connectedness graph, that is, a graph where each pair of nodes is connected to each other. The arcs are weighted by the distance between their corresponding nodes. Each graph node s∈V is modeled as an *n*-dimensional feature vector, and *w*(**s**,**t**) is a weight (distance) between two graph nodes **x**_*i*_ and **x**_*j*_ used to weight the arc 〈s,t〉∈E. Mathematically, *w* is a function that takes two graph nodes and returns the distance between them, that is, $w\to V\times V:{ℜ}^{+}$. In this work, a node is the set of *n* features extracted from a particular marmoset vocalization.

As aforementioned, the training step of the OPF classifier aims at building an optimum-path forest rooted in a set of prototype samples. Let S be that set, such that S⊆V. A very common and easy way to obtain S would be though a random sampling over the training samples. However, two requirements need to be met: (i) each class should be represented by, at least, one prototype node, and (ii) the prototype’s distribution should cover different regions of the feature space. These requirements make the naïve approach too time-consuming. In order to circumvent this problem, Papa et al. [41] proposed to place prototypes in the regions more prone to errors, that is, nearby the frontier of the classes. The idea is to compute a Minimum Spanning Tree (MST) over the training set, and then mark the connected samples from different classes as prototypes. We say that $S^{*}$ is an optimum set of prototypes when the training step minimizes the classification errors in $D_{1}$ [44]. The optimum prototypes are the closest samples of the MST with different labels in $D_{1}$.

Given the prototypes, the next step is to find the smallest path-cost from the prototypes to the remaining training samples in $S^{*}$ to the remaining nodes in $V\backslash S^{*}$. In this work, we adopted the same path-cost function as Papa et al. [41, 42], which computes the maximum arc-weight along a path, as follows:

$$\begin{array}{ccc} f_{max}\left(\left\langle s\right\rangle \right) & = & \left\{\begin{array}{cc} 0 & \text{if}\;s\in S^{*}\text{,}\\ +\infty & \text{otherwise}. \end{array}\right.\\ f_{max}\left(\pi \cdot \left\langle s,t\right\rangle \right) & = & \max\{f_{max}\left(\pi \right),d\left(s,t\right)\}, \end{array}$$ (1)

where *π* ⋅ 〈*s*, *t*〉 stands for the concatenation of path *π* and the arc 〈*s*, *t*〉. A path is defined as a sequence of distinct and adjacent samples.

After computing the optimum-path forest, unseen samples from $D_{2}$ can be classified. For each test sample, the classification step consists of connecting the sample to all training nodes, evaluating which training sample offers the optimum-path cost *C* according to *f*_*max*_ defined in Eq 1, that is:

$$C\left(t\right)=\min_{\forall s\in D_{1}}\left\{\max\left\{C\left(s\right),d\left(s,t\right)\right\}\right\}.$$ (2)

Let s* be the training sample that satisfies the above equation. Then, test sample *t* will be assigned the same label as sample s*. For the current experiments we used the LibOPF library available at https://github.com/LibOPF/LibOPF.

#### Bayesian Classifier

A Bayesian Classifier estimates the probability that a given vocalization belongs to a certain class. This probability can be derived from Bayes’ Theorem [45]:

$$p\left(\omega_{i}|x\right)=\frac{p\left(x|\omega_{i}\right)P\left(\omega_{i}\right)}{p(x)},$$ (3)

where *p*(**x**|*ω*_*i*_) denotes the probability of observing feature vector **x** given the class *ω*_*i*_, *P*(*ω*_*i*_) is the prior probability of class *ω*_*i*_, and *p*(**x**) is the probability of **x**. In order to estimate *p*(**x**|*ω*_*i*_) we assumed that the likelihood function is Gaussian, and could thus estimate its parameters from the dataset [46].

#### Multilayer Artificial Neural Network

An MLP classifier is a feedforward neural network composed of several neuron layers aiming to solve multiclass problems [47]. The input to each layer is a weighted sum of the output from the previous layer. The number of neurons in the first layer equals the number of features of the input, while the number of neurons in the last layer is equal to the number of classes. The neural network assigns a feature vector extracted from a vocalization **x** to the class *ω*_*q*_ if the *q*-th output neuron has the highest activation. We used the MLP implementation from scikit-learn [48], with two hidden layers of eight and 16 neurons. The network was trained using backpropagation and the Limited-memory BFGS optimization algorithm [49] to update the weights. The learning rate was set to 0.001.

#### Support Vector Machines

While the learning of MLP is based on the principle of empirical risk minimization, the SVM induction process is rooted in the principle of structural risk minimization [50–52], aiming at establishing an optimal discriminative function between two classes of patterns while accomplishing the trade-off between generalization and overfitting. The SVM training algorithm constructs the optimal hyperplane separating the two classes [50]. In order to extend from linear to nonlinear classification the *kernel trick* is used [51], where kernel functions nonlinearly map input data into high-dimensional feature spaces in a computationally-efficient manner.

For classification problems with multiple classes, two approaches are commonly used for binary SVMs, *one-against-one* and *one-against-all* [53]. Both strategies tend to lead to similar results in terms of classification accuracy, but the former, which was the one adopted here usually requires shorter training time, although incurring a higher number of binary decompositions.

For the current experiments, we used the LibSVM library [54] (available at http://www.csie.ntu.edu.tw/~cjlin/libsvm). The hyperparameters C and *σ* of the SVM classifier were determined via a 5-fold cross-validation grid-search in the ranges [2^−5^, 2^15^] and [2^−15^, 2^3^], changing the exponent in steps of two.

#### k-Nearest Neighbors

k-NN is a very simple algorithm that works well in many different applications. In contrast to the OPF, the k-NN uses all training samples as prototypes.

The k-NN requires an input parameter *k* setting the number of neighbors that contribute to the classification of a sample [55, 56]. In order to classify a test sample *t*, the majority label in a region (e.g. sphere or hypercube) containing *k* training samples and centered at *t*, determines *t*’s label. Note that for *k* = 1, the testing sample *t* is classified as the class of the closest training sample. For the current experiments, we defined the value of *k* as the best value of a grid-search in the range $\left[1,\left\lfloor \frac{m}{5}\right\rfloor \right]$ in steps of two; where *m* is the number of training samples.

#### Logistic regression

The logistic regression classifier is essentially a one-layer artificial neural network where the weighted input features are feed to the logistic function. Here, we trained the classifier under a one-against-all scheme, regularized by the L2 norm of the classifier weights. We used the LIBLINEAR [57] implementation of logistic regression.

#### AdaBoost

AdaBoost, short for Adaptive Boosting, is a meta-classifier that trains an ensemble of weak classifiers. It iteratively trains classifiers on the same dataset while adjusting the weights of incorrectly classified samples such that subsequent classifiers focus more on those difficult samples. The resulting ensemble classifier tends to be less susceptible to over-fitting than other classifiers, but it is also known to be sensitive to noisy data and outliers [58]. Here, we used the AdaBoost-SAMME.R algorithm [59] from the from scikit-learn package [48].

### Statistical evaluation metrics

In regard to the recognition rate, we used an accuracy measure proposed by Papa et al. [41], which is similar to the Kappa index [60], but more restrictive. If, for example, there are two classes of vocalizations with very different sizes and a classifier always assigns the label of the largest class, the average number of correct assignments will be deceivingly high. A better accuracy measure should take into account the high error rate of the smallest class. The accuracy used here is measured by taking into account that classes may have different sizes in $D_{2}$. Let us define:

$$e_{i,1}=\frac{FP_{i}}{\left|D_{2}\right|-\left|D_{2}^{i}\right|}$$ (4)

and

$$e_{i,2}=\frac{FN_{i}}{\left|D_{2}^{i}\right|}\text{,}\;i=1,2,\ldots ,K\text{,}$$ (5)

where *K* stands for the number of classes, $\left|D_{2}^{i}\right|$ concerns the number of samples in $D_{2}$ that come from vocalization class *i*, and *FP*_*i*_ and *FN*_*i*_ stand for the numbers of false positives and false negatives for class *i*, respectively. That is, *FP*_*i*_ is the number of samples from other vocalization classes that were classified as being from the class *i* in $D_{2}$, and *FN*_*i*_ is the number of samples from the class *i* that were incorrectly classified as being from other classes in $D_{2}$. The error terms *e*_*i*,1_ and *e*_*i*,2_ are then used to define the total error from class *i*:

$$E_{i}=e_{i,1}+e_{i,2}.$$ (6)

Finally, the accuracy *Acc* is then defined as follows:

$$Acc=1-\frac{\sum_{i=1}^{K}E_{i}}{2K}.$$ (7)

Sensitivity (*Se*), often called recall, is the ratio of the number of correctly classified vocalizations from a given class and the total number of vocalizations in that class (including misclassified vocalizations):

$$Se=\frac{\text{TP}}{\text{TP}\;\text{+}\;\text{FN}},$$ (8)

where *TP* and *FN* are the number of vocalizations from a given class that were correctly or incorrectly classified, respectively.

Positive predictive value (*PPV*), often called precision, is the ratio between the correctly classified vocalizations from a given class and the total number of vocalizations classified as pertaining to that class:

$$PPV=\frac{\text{TP}}{\text{TP}\;\text{+}\;\text{FP}},$$ (9)

where FP denotes the number of vocalizations incorrectly classified as belonging to the considered class.

Finally, as a more global performance metric, we calculated the averaged F_1_-score for all eight classes. The F_1_-score for a given class is calculated as the harmonic mean of the *Se* and *PPV* values for that class:

$$F_{1}-score=2\left(\frac{Se\times PPV}{Se+PPV}\right).$$ (10)

These four metrics allow us to reliably evaluate the performance of the classification algorithms considered in this work. The performance metrics are reported as the averages over 100 repetitions of classifier training and testing. All training and testing of the classification algorithms was done on a computer with an Intel i7 5500U processor with 8GB of RAM using Linux as operational system. A Python script to reproduce the results is available at https://github.com/kalleknast/call_class.

## Results

**Fig 2:**
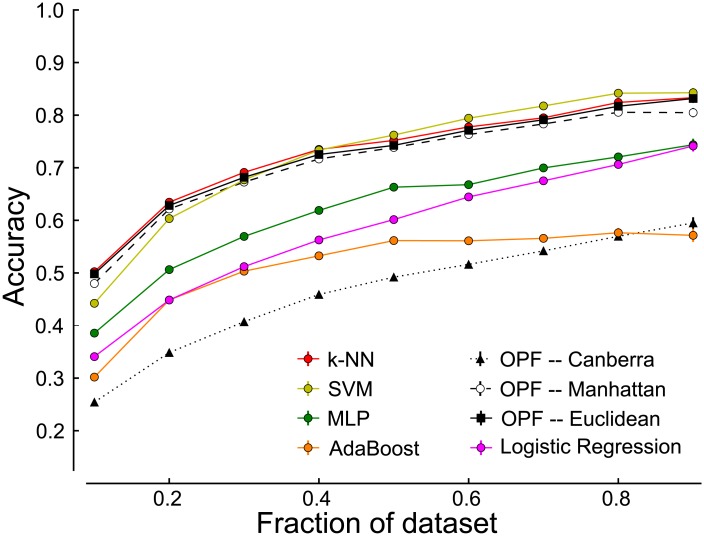
The effect of training set size on classification performance. For the sake of visual clarity, the results of OPF using the distance metrics Bray-Curtis and Chi-Square, and SVM using linear and polynomial kernels are excluded.

In order to find a robust method for the automatic classification of marmoset vocalizations, we compared the classification performance of seven different algorithms. In addition, we further explored different configurations of both OPF and SVM. OPF was tested using the following distance metrics: Euclidean, Manhattan, Canberra, Chi-Square and Bray-Curtis, and SVM was tested with linear, radial basis function and polynomial kernels. The goal was to find an algorithm that could be successfully trained on small sets of primate vocalization data. For this reason, we split our original dataset into training sets of increasing sizes, ranging from 10% to 90% of the original dataset. We found good performance of all algorithms except AdaBoost, Naive Bayes, SVM when using linear and polynomial kernels, and OPF when using Chi-Square, Bray-Curtis and Canberra distance metrics (see Table 2 and Fig 2). These poorly performing algorithms were excluded from further consideration. SVM, k-NN and OPF using Euclidean and Manhattan distances performed similarly, and well above chance (accuracy ≈ 0.5 and F_1_-score ≈ 0.5) using as little as 10% of the original dataset, and reaching an accuracy around 0.8 when trained on 90% of the data. However, in spite of the good performance, Fig 2 shows that classification accuracy keeps improving with training set size, suggesting that adding even more training data would further improve performance.

**Table 2:** Classification of all eight classes of vocalizations using different algorithms. 90% of the samples were used for the training set. Time refers to the time required to classify one sample in milliseconds.

Method	F_1_-score		Accuracy		Time (ms)
	Mean	SEM	Mean	SEM	Mean
MLP	0.757	0.010	0.744	0.011	145.6
OPF–Chi-Square	0.257	0.009	0.197	0.011	24.4
OPF–Bray Curtis	0.466	0.010	0.421	0.011	6.9
OPF–Canberra	0.622	0.010	0.595	0.011	24.5
OPF–Euclidean	0.840	0.007	0.832	0.008	7.7
OPF–Manhattan	0.818	0.008	0.805	0.009	10.7
k-NN	0.842	0.007	0.833	0.008	465.1
SVM	0.852	0.008	0.843	0.009	115.2
Naive Bayes	0.507	0.010	0.475	0.011	252.0
AdaBoost	0.603	0.011	0.571	0.013	10173.1
Logistic regression	0.744	0.010	0.741	0.011	89.6

The OPF algorithm configured with the Euclidean distance metric was selected for further analysis since it yielded better or comparable classification performance on both the smallest and the largest datasets (top accuracy ≈ 0.83 and F_1_-score ≈ 0.84). It is parameter free and thus, easy to train, and computation time is an order of magnitude less than for SVM and k-NN (see Table 2). Table 3 presents the results as a confusion matrix.

**Table 3:** Confusion matrix considering the classification of all eight classes of vocalizations using OPF with Manhattan distance and 90% of the samples for training set.

		**True class[%]**							
		**Alarm**	**Chirp**	**Loud-shrill**	**Phee**	**Seep**	**Trill**	**Tsik**	**Twitter**
**Classified as[%]**	**Alarm**	78.4	0.0	4.7	4.1	4.3	0.7	2.5	0.0
	**Chirp**	3.9	89.5	0.0	1.6	0.0	0.0	4.4	7.0
	**Loud-shrill**	5.9	0.0	75.3	5.9	0.0	19.2	0.0	0.4
	**Phee**	4.3	0.1	4.3	83.0	0.0	0.9	0.0	0.5
	**Seep**	5.7	0.0	0.0	0.0	90.6	0.0	8.3	0.0
	**Trill**	1.6	0.0	15.7	0.0	0.0	74.0	0.0	0.0
	**Tsik**	0.2	1.0	0.0	0.0	5.1	0.0	77.5	0.0
	**Twitter**	0.0	9.4	0.0	5.4	0.0	5.2	7.3	92.1

We opted for a hierarchical strategy to investigate how well all 11 types of vocalization in the dataset could be classified. Such approach was performed through sorting the Phee and Tsik classes into three and two sub-classes, respectively. The Phee class was sorted into Phee-2, Phee-3 and Phee-4 depending on how many separate whistles the call contained (Fig 1(d), 1(e) and 1(f), respectively). The Tsik call was sorted into the sub-classes Tsik and Tsik-ek depending on the presence of the harmonic “ek” component following the “tsik” in the Tsik-ek calls [37] (Fig 1(i) and 1(j), respectively).

Table 4 presents a confusion matrix of the results from the Phee calls using OPF with Euclidean distance metric. Overall, the classification accuracy (56%) was not quite as good as when classifying the eight principal classes. Phee-2 was correctly classified in 70% of the cases, and Phee-3 was classified at 46%, where the remaining 64% were misclassified as Phee-2 15% or Phee-3 39%. Finally, Phee-4 was accurately classified in 52% of the samples, where the remaining 48% were misclassified as Phee-3 34% or Phee-2 15%. Classification of the Phee sub-class from the eight principal classes resulted in a compounded accuracy of 58% for Phee-2, 38% for Phee-3, and 42% for Phee-4, since accuracy for the principal Phee class was 83% (see Tables 3 and 4).

**Table 4:** Confusion matrix considering the classification of the principal Phee class into sub-classes using OPF with Euclidean distance metric and 90% of the samples for training set.

		**True class[%]**		
		**Phee-2**	**Phee-3**	**Phee-4**
**Classified as[%]**	**Phee-2**	70.4	19.8	9.7
	**Phee-3**	14.8	46.2	39.1
	**Phee-4**	14.8	34.0	51.2

Table 5 presents a confusion matrix of the results using OPF with Euclidean distance metric for Tsik vocalizations. The overall accuracy was 83%, where the accuracy for sub-class Tsik was 91%, and that of Tsik-ek was 76%. The compounded accuracies for the sub-classes Tsik and Tsik-ek were 70% and 58%, respectively (see Tables 3 and 5).

**Table 5:** Confusion matrix for the classification of the principal Tsik class into sub-classes using OPF with Manhattan distance metric and 90% of the samples for training set.

		**True class[%]**	
		**Tsik**	**Tsik-ek**
**Classified as[%]**	**Tsik**	90.5	24.5
	**Tsik-ek**	9.5	75.5

## Discussion

In this work, we presented methods for the automatic classification of commonly occurring vocalizations of the common marmoset. The provided dataset can be used for acoustic analysis, further algorithm development and playback experiments. The method presented should enable the online monitoring of vocal activity in colonies of captive marmosets, so as to provide valuable information about the colony’s health and well-being. Further, the method allows for interactive experimental designs, in which different actions can be triggered depending on the vocal behavior of the subjects.

Since recording and manually labeling data is both labor-intensive and time-consuming, the most important factor when selecting a classification algorithm is how well it performs on a small amount of data. Fig 2 demonstrates clear advantages for the k-NN, SVM and OPF (using Euclidean and Manhattan distances) algorithms. These algorithms perform best on both the least and the greatest amount of data.

Another factor to consider is how easy an algorithm is to use. Most algorithms have hyperparameters that require careful optimization for good performance. The standard way of doing this is to repeatedly re-train the algorithm on a manually specified range of hyperparameter values, each time evaluating classification performance on data that were not included among the training data. Both the k-NN and SVM algorithms require such hyperparameter optimization, whereas the OPF algorithm is parameterless, making it easier to use.

Under some circumstances, the time required for classification can become important. Algorithms that do not require much computational resources are valuable when real-time classification is necessary, and especially when the computations are performed on small single board computers or embedded devices with limited capacity. Examples of this are on-site portable audio acquisition and analysis [61], or home-cage vocal conditioning systems [62]. The OPF algorithm requires an order of magnitude less time than comparably performing k-NN and SVM algorithms and is thus suitable for these goals.

The proposed methods are mainly suited for laboratory conditions where audio can be recorded under consistent conditions, and with high signal-to-noise ratio. Such conditions are unlikely to be available under field conditions. Robust classification within field conditions would probable require much improved pre-processing before the classification step.
